# Suppression of established hepatocarcinoma in adjuvant only immunotherapy: alum triggers anti-tumor CD8^+^ T cell response

**DOI:** 10.1038/srep17695

**Published:** 2015-12-09

**Authors:** Bo Wang, Xuanyi Wang, Yumei Wen, Jing Fu, Hongyang Wang, Zhangmei Ma, Yan Shi, Bin Wang

**Affiliations:** 1 Key Laboratory of Molecular Medical Virology, MOE/MOH, Shanghai Medical College, Fudan University, Shanghai, China; 2 Institute of Biomedical Sciences, Fudan University, Shanghai, China; 3 International Cooperation Laboratory on Signal Transduction, Eastern Hepatobiliary Surgery Institute/Hospital, and National Center for Liver Cancer, Shanghai, China; 4 Institute of Immunology, Department of Basic Medical Sciences, Center for Life Sciences, Tsinghua University, Beijing, China; 5 Department of Microbiology, Immunology and Infectious Diseases, University of Calgary, Calgary, Canada

## Abstract

Dendritic cell-based immunotherapy is a new weapon in our battle against malignancies in human. Recent trials in human and research work in model animals have shown various degrees of success, suggesting its great potential for clinical use. While protocols vary, a common scheme in this category of treatment involves activation of dendritic cells, with the purpose of increasing antigen presentation and cellular immunity. Therefore, proper use of immune adjuvant is a central subject of study. We report here an unexpected finding that injection of alum, the most widely used human adjuvant, into mice carrying H22 hepatocarcinoma resulted in a significant reduction of tumor growth with extended animal survival. This effect was associated with an increased specific CD8^**+**^ T cell activation and an inflammatory environment, yet with minimal overt side effects. Our finding suggests that use of adjuvant alone in certain established tumors can invoke protective host immune activation against the same target, which may be of value in our development of new cancer immunotherapies.

Immunotherapy of cancer has been regarded as one of the biomedical breakthroughs in recent years^1^. The goal of immunotherapy is to invoke host immune responses to control and in optimal cases to eradicate the neoplasm, which in contrast to conventional tumor treatment is safe with fewer side effects. Currently there are over one thousand clinical trials under this category being carried out^2^ (data extracted from www.clinicaltrials.gov). Among them, adoptive cell transfer (ACT), immune checkpoint blockage and dendritic cell-based vaccines are most intensely studied^3^,^4^,^5^.

Unlike prophylactic vaccination whereby host immune response is induced in preparation of future encounters of infectious agents, cancer immunotherapy is to break the state of tolerance towards antigens errantly present or overly expressed in tumor cells^6^. ACT involves *in vitro* expansion of host T cells stimulated by tumor antigens, in the absence of *in vivo* inhibitory factors, and reinfusion of these cells into the host for cytolysis and apoptosis induction of the tumor^7^,^8^. More recent efforts apply biomedical engineering technologies through which tumor antigen- specific receptors are expressed on the infused lymphocytes for more robust recognition^9^,^10^. Immune checkpoint blockage takes advantage of some common tactics used by cancerous tissues to shield themselves from immune detection, particularly via signaling of cell surface negative immune regulators. Antibodies against CTLA-4 have been used successfully in treating metastatic melanoma^11^,^12^,^13^. Blocking PD-1/PD-L1 signaling has also shown great efficacy in treating papilloma virus-induced malignant lesions and a list of other solid tumors^3^,^14^. While these protocols hold great potential, they are not without peril. ACT suffers from difficulty in antigen identification and technical challenges in immune cell expansion^15^,^16^, check point blockage is only applicable in a limited number of solid tumors^10^ and is often associated with autoimmunity, including colitis and dermatitis^17^,^18^. DC-based immune therapy, which aims at increasing the intensity and breadth of antigen presentation, remains a valid alternative.

Dendritic cells constantly present host endogenous antigens to T cells that in the absence of danger signal serves as a mechanism of peripheral tolerance induction^19^. Tumor antigens are presented in this context. In the tumor environment, additional negative regulations are often present, including tumor-associated macrophages and suppressive cytokines such as TGFβ^20^,^21^,^22^. In this case, adjuvant becomes critically important in triggering activation of DCs^23^. Effectuating through TLRs/NLRs, phagocytosis induction, or DC membrane alteration, adjuvants often induce strong DC activation, leading to robust antigen presentation, expression of costimulatory molecules and secretion of inflammatory cytokines^24^,^25^,^26^. DC-based vaccines can be roughly divided into three categories. DCs isolated from the host or/and expanded *in vitro* can be loaded with tumor antigens (epitope peptides or autologous tumor lysates) in the presence of adjuvant, and reinfused into the host^27^,^28^. A more targeted approach uses tumor cells that are engineered to express GM-CSF to specifically attract DCs *in vivo*^29^. More recently, tumor antigens have been fused to antibodies that specifically recognize DC surface markers, such as DEC205, DNGR1, CD40 etc. for better targeting, often achieving immune response in the absence of additional adjuvant^30^,^31^,^32^. The most basic/passive protocol uses tumor antigens admixed with adjuvant in hopes that DCs would capture and present those antigens upon stimulation^33^,^34^. Conceptually, since antigens from established tumors are constantly presented by DCs, leading to immune tolerance in the absence of DC activation, proper stimulation of DCs may in theory reverse the inhibition and invoke tumor immunity.

We report here an unexpected finding that in Balb/c mice with an established H22 hepatocarcinoma, a protocol of repeated alum injections invoked a tumor-specific immune response that significantly inhibited tumor growth and mortality. This response was critically dependent on the adoptive immune system, particularly CD8^**+**^ T cells, and to a lesser extent neutrophils. Importantly, this protocol triggered little systemic inflammation and tissue damage. Our results therefore suggest that administrating adjuvant alone in tumor-bearing hosts may lead to tumor suppression, likely via nonspecific activation of DCs. Since alum is a well-tolerated adjuvant, our outcomes therefore implicate its potential use in tumor treatment.

## Results

### A specific protocol to induce anti-tumor effect by alum-only therapy

**Figure 1:**
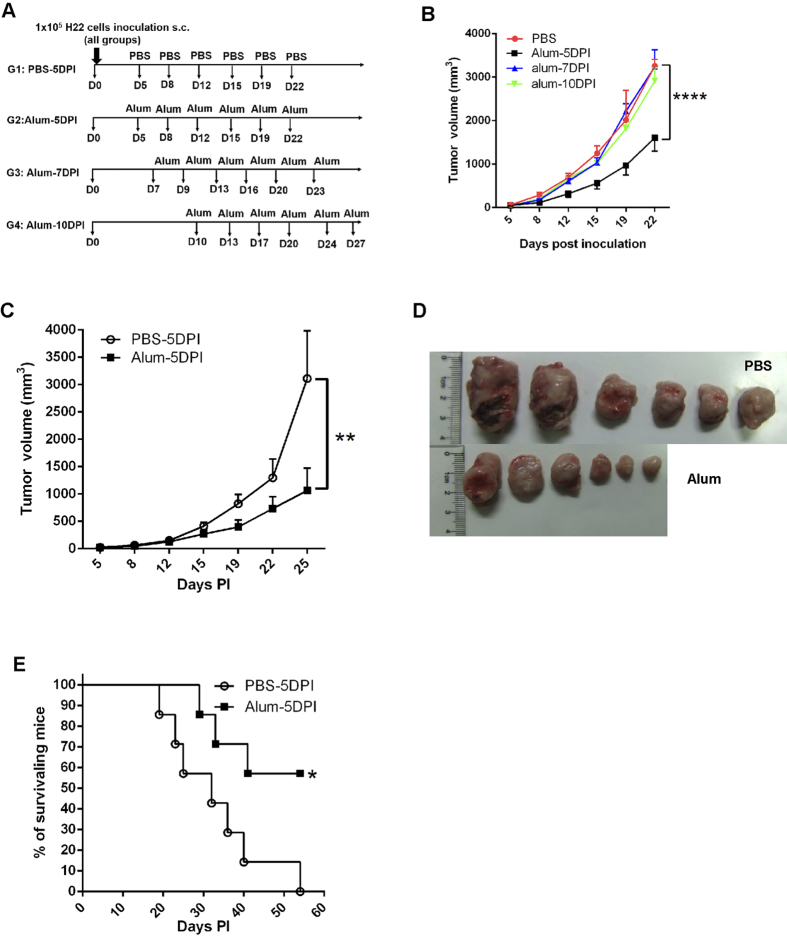
The alum alone treatment inhibits H22 tumor growth. Groups of female Balb/c mice (seven per group) were inoculated s.c. with 10^5^ per mouse of H22 tumor cells on day 0. Tumor-bearing mice were injected i.p. with 0.25 mg/250ul Al(OH)3 or 250 ul PBS, twice weekly for 3 consecutive weeks. (**A**) A schematic representation of treatment protocol. (**B**) Line graph depicting the tumor volume in H22 tumor-bearing mice receiving different treatments over time. (**C**) Tumor volume changes in H22 tumor-bearing mice treated with Al(OH)3 or PBS starting on the 5th day. (**D**) Photos of tumors from mice sacrificed and solid tumors isolated on day 22. (**E**) Kaplan-Meier survival analysis of each group. Each value represents mean ± SEM. *p < 0.05, **p < 0.01, and ****p < 0.0001. Unmarked comparisons are not statistically significant.

In our attempts to observe alum as an adjuvant to boost anti-tumor response against a Balb/c hepatoma line H22 initially established from a lymphatic metastatic model^35^, we immunized H22 tumor-bearing mice with various isogenic tumor cell lysates in combination with alum to detect any potential therapeutic effect. We noticed an occasional reduction of tumor size in alum alone group in the absence of tumor antigen (tumor lysate). To establish a protocol to capture this effect more consistently, we set up a series of experiments to identify the optimal immunization schedule. A representative scheme is outlined in Fig. 1A. H22 hepatocarcinoma cells were first inoculated i.p. in Balb/c mice, the peritoneal lavage was collected and 10^5^ cells from the lavage were injected s.c. into new recipients. 5, 7 or 10 days later, 250 μg of Al(OH)3 in 250 μl of PBS or PBS alone was injected i.p. into the inoculated mice, followed by the same treatment every three or four days for a total of 6 injections (Fig. 1A). In all schedules of alum treatment, those initiated 7 or 10 days post tumor inoculation showed tumor volume increase similar to the PBS control. Interestingly, the treatment initiated 5 days after (alum 5DPI) exhibited significant growth reduction, with the differences becoming greater over time (Fig. 1B). We then selected this schedule for more detailed analyses. In line with the initial finding, tumors removed from the alum 5DPI-treated mice 25 days after the inoculation were visibly smaller than the PBS control (Fig. 1C,D). This was accompanied by a statistically significant survival advantage as no mice in the PBS group remained at day 54, in contrast with a 50% survival rate in the alum 5DPI group (Fig. 1E). Although *in vivo* growth potential of H22 changed over time likely as a consequence of cell culture which resulted in slightly different growth rates from experiment to experiment, this outcome nonetheless suggests that alum injection alone, administered after hepatocarcinoma establishment, in comparison with PBS control leads to an unexpected suppression on tumor growth.

### Enhanced immune activation in alum 5DPI-treated tumor-bearing mice

**Figure 2:**
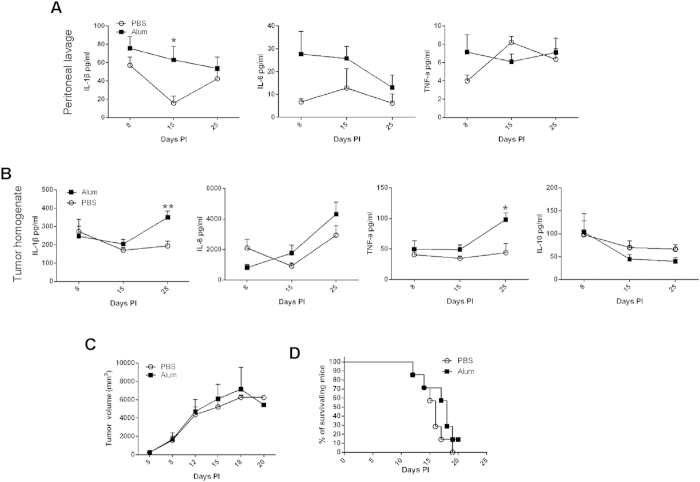
Enhanced immune activation in alum 5DPI-treated tumor-bearing mice. Balb/c mice were inoculated s.c. with 10^5^ H22 cells and randomly divided into two groups 5 days later. Mice were treated with Al(OH)3 as indicated. Peritoneal wash fluid and tumor interstitial fluid were collected 3 days after the 1st, 3rd, and 6th alum injection. Levels of IL-1β, IL-6, TNF-α and IL-10 in peritonea (**A**) and tumor (**B**) were analyzed by ELISA. (**C**) Nude mice were inoculated with 10^5^ H22 cells and treated with Al(OH)3 or PBS. Line graph depicting the tumor volume in H22 tumor-bearing nude mice receiving alum or control treatment. (**D**) Kaplan-Meier survival analysis of H22 tumor-bearing nude mice. Each value represents mean ± SEM. *p < 0.05, **p < 0.01. Unmarked comparisons are not statistically significant.

To probe the immunological changes associated with the tumor suppression in Fig. 1, we followed cytokine profile in peritoneal lavage and tumor homogenate over time. Figure 2A shows that IL-1β and IL-6 were slightly elevated in the lavage of the alum 5DPI mice in comparison to the PBS control, although the absolute quantities were quite low. In contrast, amounts of IL-1β and TNFα were increased in the alum-treated groups (Fig. 2B), suggesting the treatment is associated with an enhanced inflammatory response.

Alum was administered spatial-temporally away from the H22 tumor inoculation. By default, the presence of particulate or crystalline structures can activate innate immune responses, particularly as a consequence of interaction with phagocytes. Whether the alum-mediated tumor suppression also required the adaptive immunity was of great interest. To that end, we carried out the experiment of Fig. 1 in nude Balb/c mice. Figure 2C,D show that the protective benefit wrought out by alum was lost in the absence of cellular immunity, as assessed by tumor size or mortality.

### CD8^**+**^ T cells are essential to alum-associated tumor suppression

**Figure 3:**
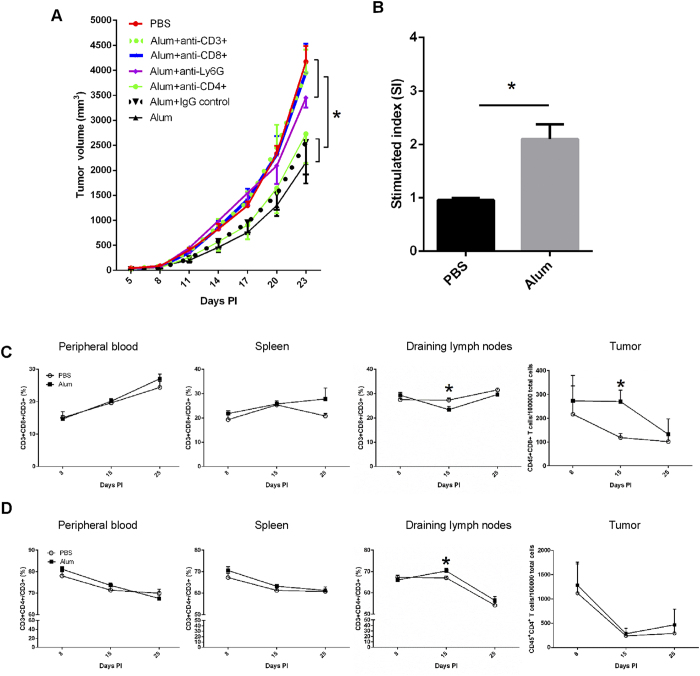
CD8^+^ T cells are essential for alum-induced tumor suppression. (**A**) Balb/c mice were inoculated s.c. with H22 cells and treated with Al(OH)3 or PBS. Tumor-bearing mice were injected i.p. with 100 μg of anti-Ly6G, anti-mouse CD8 or rat IgG2a κ isotype control antibody in 200 μl PBS 1 day after each Al(OH)3 injection. The tumor volume was measured every 3 days. (**B**) Draining LNs were harvested from tumor-bearing mice in PBS- and Al(OH)3-treated groups 15 days after tumor inoculation. Single cell suspensions were prepared, labeled with cell proliferation dye eFluor 670 and stimulated with 10 μg/ml H22 cell lysis (protein) for 72 h and analyzed by FACS. Peripheral blood, spleen, draining LNs and tumor were harvested from tumor-bearing mice 3 days after the 1st, 3rd, 6th injection. The percentages of CD8^+^ (**C**) and CD4^+^ (**D**) T cells in total T cells or in total cells were analyzed by FACS. P values for D15 CD8^+^ and CD4^+^ cells in draining lymph nodes and CD8^+^ cells in tumor are 0.035, 0.045, and 0.047 respectively.

The control over neoplastic pathogenesis by the cellular immunity is highly complex. While CD8^**+**^ T cells can exert their effect via cytolysis and apoptosis induction, CD4^**+**^ T cells are increasingly recognized as an important factor as well, modulating cytokine production and tumor microenvironment^36^, in addition to a subset of these cells, regulatory T cells, serving as a balancing factor. On day 6, 9, 13, 16, 20, and 23 after inoculation, we i.p. infused blocking antibodies against CD8, CD4 and neutrophil marker Ly6G into mice treated with alum 5DPI. Figure 3A shows that depletion of CD3^**+**^ or CD8^**+**^ T cells essentially eliminated the protective effect; while CD4^**+**^ T cell removal produced a marginal, statistically insignificant reduction in protection. Neutrophil depletion also resulted in an intermediate reversal of protection. To confirm the role of CD8^**+**^ T cells, lymphocytes from alum 5DPI-treated groups were harvested after three injections, and stimulated with H22 tumor lysate. The expansion of CD8^**+**^ T cells was analyzed by FACS and the division index (ratio of expansion in the presence over in the absence of tumor lysate) was plotted in Fig. 3B. Clearly, CD8^**+**^ T lymphocytes in the alum-treated mice showed substantial expansion, in comparison with the PBS control. While overall the percentage did not change in the blood, even slightly decreased in LNs that drained the site of inoculation, large numbers of CD8^**+**^ T cells infiltrated the tumor in the alum group (Fig. 3C). In keeping with the lesser involvement of CD4^**+**^ T cells, no change was seen for CD4^**+**^ T cells at these locations (Fig. 3D). Therefore, alum-treated mice show enhanced H22 tumor-specific CD8^**+**^ T cell immunity, while CD4^**+**^ T cell response does not appear to be critical. We failed to detect significant NK cells in the tumor (near or below 1%). On the other hand, Treg cells appeared in higher number on day 15 in PBS control in comparison with alum treated group, suggesting that alum might suppress the increase of Treg cells in tumor growth environment (Supplemental Fig. 1).

### Substantial changes in gene regulation and chemokine production following alum treatment

**Figure 4:**
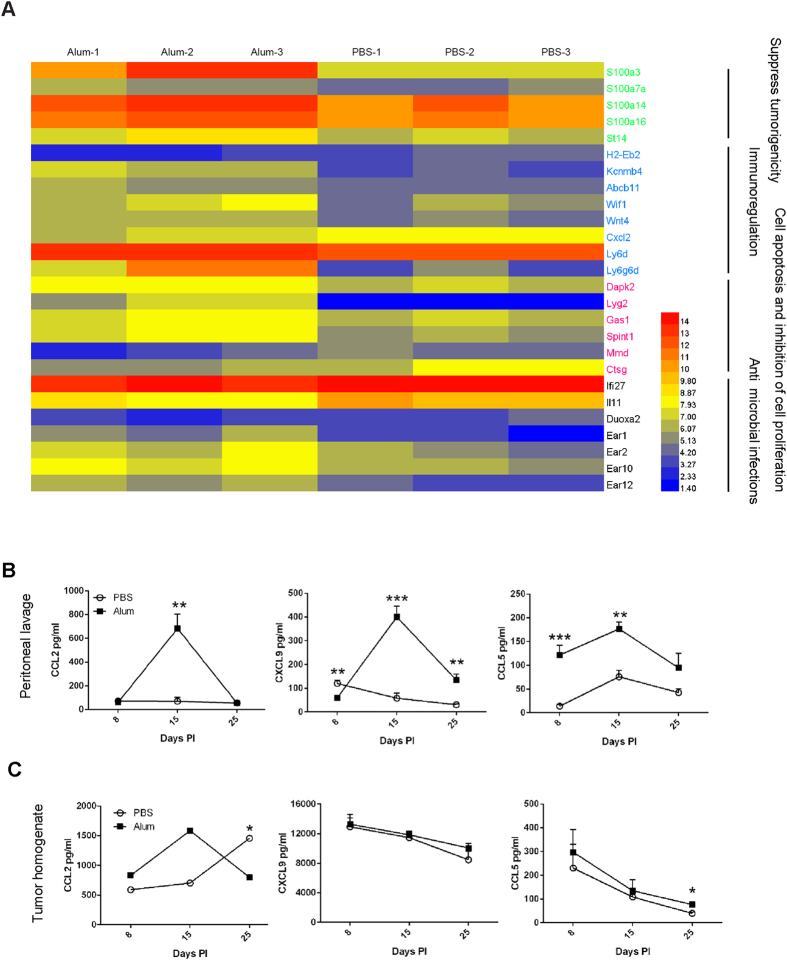
Changes in gene regulation and chemokine production following Al(OH)3 treatment. (**A**) Heat maps showing the hierarchical clustering of samples along genes in a fixed order for tumor sample in PBS- and Al(OH)3-treated groups. Tumors were harvested from tumor-bearing mice 15 days after the inoculation. Total RNA was extracted and quantitated as described in the methods. The comparison between two groups was carried out using three biological replicates. Shown are transcripts classified as tumor suppression-related and those expression levels significantly different between two groups. (**B**,**C**) Balb/c mice were inoculated with10^5^ H22 cells, 5 days later, tumor-bearing mice were randomly divided into two groups. Peritoneal wash fluid and tumor interstitial fluid were collected from mice 3 days after the 1st, 3rd and 6th injection. CCL2, CXCL9 and CCL5 in peritonea (**B**) and tumor fluid (**C**) were analyzed by ELISA. Each value represents the mean ± SEM. *p < 0.05, **p < 0.01, ***p < 0.001. Unmarked comparisons are not statistically significant.

To globally analyze how the alum treatment impacts H22 tumor, a gene expression profile of surgically recovered tumor 15 days after inoculation was studied by microarray genechip analysis. After alum treatment there were 364 genes differentially expressed (Supplemental Fig. 2). Among these genes, 20 were up-regulated. All these genes can be divided into four categories: 1. suppression of tumorigenicity, such as St14 (Suppression of Tumorigenicity 14)^37^; 2. immune regulation, such as Wif1 (Wnt Inhibitory Factor 1)^38^; 3. apoptosis induction and proliferation inhibition, such as Dapk2 (Death-Associated Protein Kinase 2)^39^, Gas-1(Growth Arrest Specific 1)^40^; 4. anti-microbial infections, such as Ear2 (Eosinophil-Associated Ribonuclease 2), 10, 12, and 4B^41^,^42^ (Fig. 4A). Although the immediate impact of these gene regulations was not clear in alum-treated mice and factors such as chemokine changes were not detected (see below), this assay nonetheless showed a different profile suggestive of tumor suppression in those mice.

We reasoned that infiltration of CD8^**+**^ T cells into the tumor was the result of chemotactic changes. Peritoneal lavages and lysates of recovered tumor were monitored for CCL2, CCL9 and CCL5 levels. Figure 4B shows that all these chemokines were present in greater amounts in alum treated group, at least on day 15 after the tumor inoculation. Interestingly, only CCL2 was found to be elevated in tumor lysate on day 15, in line with the ability of this chemokine to attract CD8^**+**^ T cells (Fig. 4C). Since it appeared quite late, this increase suggested a heightened monocytic infiltration concomitant with an increased CD8 response towards the end of treatment schedule.

### Neutrophils enter the tumor

**Figure 5:**
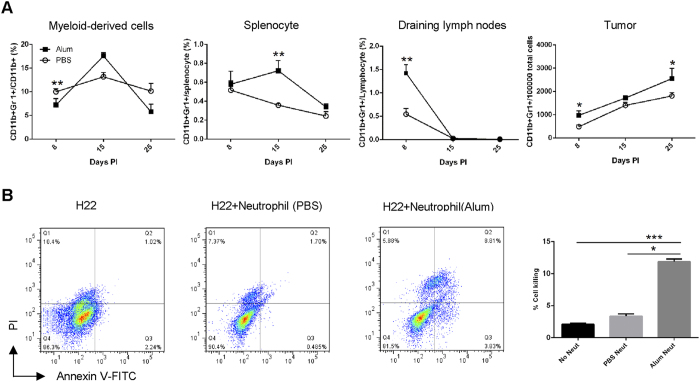
Neutrophils in tumor. (**A**) Mice with established tumors were injected i.p. with Al(OH)3 or PBS. Peripheral blood, spleen, draining LNs and tumor were harvested from tumor-bearing mice on day 3 after the 1st, 3rd and 6th injections. The percentage of neutrophils in myeloid-derived cells or total cells of spleen, draining LNs and tumor were detected by FACS. (**B**) Neutrophils were purified from tumor-bearing mice in different groups, and cocultured with eFluor 450-labeled H22 cells at ratio of 20:1. 12 hour later, tumor cells were gated and their apoptosis was evaluated (right chart). (**C**) The percentages of tumor cells killed were calculated. Each value represents the mean ± SEM. *p < 0.05, **p < 0.01, ***p < 0.001. Unmarked comparisons are not statistically significant.

In Fig. 3A, Ly6G antibody treatment led to a perceivable reverse of alum’s anti-tumor effect. Unlike tumor antigen-specific CD8^**+**^ T cells, the association of neutrophils and tumor is more complex. While it is generally believed that these cells help shape a pro-growth microenvironment thus promoting tumor, more recent studies suggest that they in some cases also kill tumor cells^43^,^44^,^45^. In the alum-treated group, there was little change of overall neutrophil numbers in the blood, however, they were elevated in the tumor, suggesting that alum is conducive to a pro-inflammatory environment (Fig. 5A). To see if the recruited cells were more efficient in inducing tumor cell death, neutrophils recovered from the blood were used in a co-culture assay with H22 cells. Clearly, neutrophils from the alum-treated mice were much more efficient in mediating Annexin V turnover on the target cells than ones from the PBS group (Fig. 5B), in line with the reduced tumor suppression when Ly6G antibody was used to deplete this population.

### No adverse tissue damage following alum injection

**Figure 6:**
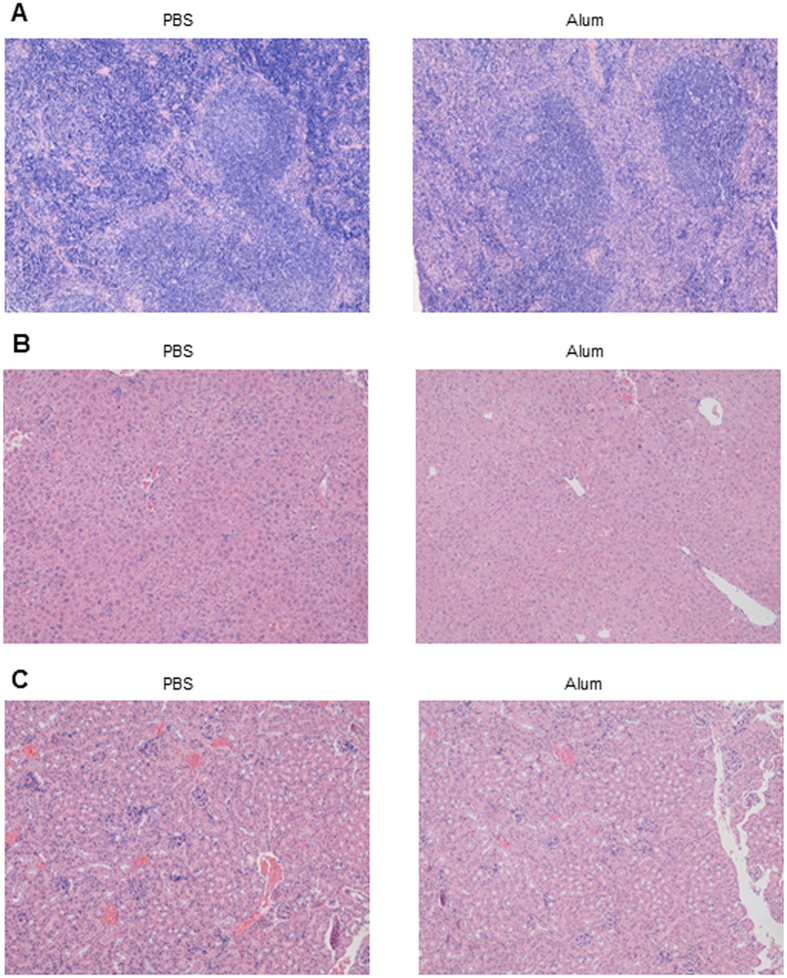
Lack of general damage in mice treated with alum. Mice with established tumors were injected i.p. with Al(OH)3 and PBS. Spleen, liver and kidney were excised from mice 3 days after the last treatment, sectioned and evaluated by H&E staining (Magnification ×100). (**A**) Spleen from PBS- or alum- treated mice; (**B**) Liver from two groups; (**C**) Kidney from two groups.

In the absence of co-administered antigen, alum presumably exerts it anti-tumor effect by nonspecifically upregulating antigen presentation, to activate antigen-specific T cells. Without targeting specific antigens, it is possible that immune responses may be induced against a plethora of epitopes derived from tumor antigens, including those normally present on regular host cells. This could lead to autoimmunity. On the other hand, since in our protocol alum was administered repeatedly, its ability to provoke innate immunity could induce unintended tissue damage. Previous figures show that the overall immune cell numbers or cytokine levels were not drastically changed in the blood and the spleen, suggesting the lack of systemic damage. Spleen, kidney and lung sections were prepared following the full course of alum 5DPI treatment. H&E staining did not show any gross difference between samples from the PBS- and the alum-treated mice (Fig. 6A–C), confirming that repeated alum treatment is likely a safe protocol.

## Discussion

First used in human by Glenny in 1920’s^46^, extended to population-based polio vaccination by Salk in 1952^47^, alum has been the most widely used adjuvant. Detailed composition of alum, broadly defined as trivalent aluminum salt, has changed over time, from the original crude aluminum potash, to the predominant Al(OH)3 and AlPO4 in use now. Alum has been proven safe and is efficient in inducing antibody response, particularly IgG1 and IgE^48^. Its inability to trigger TH1/CTL responses, however, remains its main deficit^49^. Due to the pressing demand to develop vaccines to combat tumor and viral infection, strong efforts have been made to increase its ability to induce crosspresentation and CD8 T cell activation. For instance, alum has been mixed with a LPS-derivative lipid A to increase response against malaria^50^. Saponin-based QS21 has also shown potential to induce CD8 T cell activation^51^. While most TH1/CD8 adjuvants are too toxic for human use, those suitable for population-based immunization are still in development or in limited use. This report therefore represents a surprising finding that injection of alum with a carefully designed protocol may induce anti-tumor CTL response. It is important to note that alum, an adjuvant which has been safely used for near a century, shows non-specific tumor suppression effects by triggering CD8 T cell response. Although the response was mild in comparison with other protocols and requires meticulous precision in timing, its implications in future development of adjuvant-alone tumor therapy are intriguing.

Despite its long history, the mechanism of alum’s adjuvanticity is still a topic of debate. In early years, alum was used to precipitate microbial antigens (toxoid). It was therefore believed that it functioned as a depot against diffusion^52^. However, in mice deficient in fibrinogen that is essential for alum to form nodular structures at the site of injection, the efficacy of alum does not change^53^. In addition, surgical removal of injection site hours after the inoculation does not affect its adjuvanticity^54^. The depot theory was therefore discarded. In the last decade, alum has been found to stimulate phagocytes (macrophages and DCs) to secrete IL-1β by activating NLRP3 inflammasome^55^,^56^. This was followed by a report suggesting that NLRP3 was critical for alum’s adjuvant effect^57^. This notion has been challenged by multiple follow-up reports in which NLRP3/Caspase-1 deficiency was not found to reduce alum-induced antibody production^24^,^58^,^59^,^60^,^61^,^62^,^63^. More recently, we have reported that alum crystals were able to sort DC plasma membrane lipid species, mainly by its strong binding to sphingomyelin. This lipid rearrangement induced a Syk-dependent PI3K signaling, leading to DC activation^64^. Crystalline structures in tissues often lead to cell death and release of intracellular contents. Two reports suggested that DNA release from dead cells may be captured by DCs, and activate these cells in MHC II/TLR9 dependent manner^24^,^65^. In addition, alum has also been shown to trigger DC secretion of PGE2^26^,^66^, a known DC activating factor^67^,^68^. It is interesting since both PI3K and PGE2 are inhibitors of antigen presentation^66^,^69^. Our finding that alum can trigger CD8^**+**^ T cell activation appears to disagree with these reports. However, alum-treated mice also produce uric acid^61^,^70^, the resulting MSU crystal is a strong activator of crosspresentation^71^,^72^. Germain’s group reported nearly 20 years ago that presence of a phagocytic target on the surface of macrophages drove the external antigens into the crosspresentation pathway^73^,^74^. We recently suggested a potential mechanism responsible for this redirected antigen presentation: binding of phagocytic targets slows down the endocytic maturation in DCs. The external antigens entering the cell through this pathway are delayed in their processing for MHC class II antigen presentation. Instead, the antigens are redirected to cell surface in complex with MHC class I molecule via endocytic recycling pathway^75^.

The surprise of this work is the lack of tumor suppression in alum injection schedules other than 5DPI. In our previous human clinical trials and mouse model work, we reported that multiple injections of alum were necessary to induce a protective effect targeting HBV HBeAg or inflammatory responses, respectively^76^,^77^. Therefore the lack of effect in the other schedules might reflect that alum 5DPI captured a window in tumor establishment penetrable to immune surveillance, which would disappear at later time points. It remains elusive how exactly alum injected i.p. resulted in better antigen presentation and CD8^**+**^ T cell/neutrophil recruitment to the tumor. It is possible that alum injected at a distal site may promote better inflammatory DC conversion from circulating monocytes^78^,^79^ which might later migrate to the site of inoculation. In addition, alum is an amorphous cluster of crystals, with some sizes in the nanometer range able to travel long distance to other sites of the body^80^. Upon arrival of these DCs or crystals to the tumor, a milieu that favors antigen presentation, particularly with the assistance of simultaneously induced proinflammatory cytokines, can be established^81^. However, clarification of these details awaits further analyses.

Specific DC targeting in anti-tumor vaccination has its advantage in potency. However, it is important to recognize that in a full scale tumor-specific immune response, a strong CD8^**+**^ T cell activation relies more than DCs alone. Other cell types, such as CD4^**+**^ T, NK, and neutrophils also participate for the final outcome^82^. In addition, DCs are comprised of multiple subtypes and a given DC targeting method likely fails to reach some populations that are potentially important in tumor immune responses^83^,^84^. Injection of alum alone in tumor-bearing mice is a passive and outcome-based strategy that bypasses the full assessment of various cell types and activation pathways. With its record of safety, this simplistic tumor immunotherapy may lead to additional adjuvant alone treatments to probe their potential use in human patients.

## Methods

### Animals and reagents

Female Balb/c mice were purchased from Shanghai SLAC Laboratory Animal Co. LTD. Female nude mice (5- to 6-week-old) were obtained from Shanghai Institute of Materia Medica (Chinese Academy of Sciences, Shanghai, China). All mice were kept under specific pathogen-free conditions at Fudan University and handled according to the animal welfare guidelines of Fudan University. Mouse hepatoma H22 cells were kindly donated by Hengrui Zhang (Peking University Hepatology Institute, Beijing, China). H22 cells were cultured in complete media consisting of RPMI1640, 100 U/ml penicillin, 100 μg/ml streptomycin, and 10% fetal bovine serum (FBS).

### Ethics statement

All experimental methods involving mice were approved by the Animal Protocol Committee of Fudan University and conducted in accordance with the guidelines set forth by Institutional Animal Care and Use Committee (IACUC).

### Antibodies and fluorescent dyes

Fluorescent-labeled rat anti-mouse monoclonal antibodies: anti-CD3e-FITC (145-2C11), anti-CD4-FITC (GK1.5), anti-CD4-APC (GK1.5), anti-CD8α-PE (53-6.7), anti-CD11b-FITC (M1/70), isotype controls, functional grade purified monoclonal antibodies for depletion of neutrophils, CD3^**+**^ , CD4^**+**^ , CD8^**+**^ T cells including anti-mouse Ly6G (Gr-1) (Rb86-C5), Rat IgG2b κ isotype control (eB149/10H5), anti-mouse CD3e (145-2C11), anti-mouse CD4 (GK1.5), anti-mouse CD8 (53-6.7), Cell Proliferation Dye eFluor 670 and Cell Proliferation Dye eFluor 450 were purchased from eBioscience. Anti-mouse-Ly6G-Pacific Blue (1A8) was purchased from Biolegend.

### *In vivo* tumor treatment experiment

H22 cells were transferred into the peritoneal cavity of a Balb/c mouse. 7 days later, the ascites were harvested and diluted with PBS to a concentration of 10^6^ cells/ml. A total of 10^5^ H22 cells in 0.1 ml were s.c. injected into the right flank of Balb/c mice. After 5 days to allow tumor establishment, the tumor-bearing mice were randomly divided into four groups (5 per group): Group 1 was i.p. injected with PBS starting on the 5th day; Group 2 with Al(OH)3 on the same day; Group 3 with Al(OH)3 starting on the 7th day; and Group 4 with Al(OH)3 starting on the 10th day. For alum injection Balb/c mice (7 per group) were i.p injected with Al(OH)_3_ (National Vaccine & Serum Institute, Beijing, China) at a dose of 0.25 mg twice a week for up to 3 weeks. The given doses were diluted with PBS solution to 1 mg/ml (250 μl). Tumor growth was measured using a caliper every 3 days and the sizes were recorded as the greatest longitudinal diameter (length) and the greatest transverse diameter (width). Tumor volume was calculated by the following equation: Volume = 0.52 × length × width^2^. Mice were sacrificed when tumor reached 20 mm in length. For tumor inoculation in nude mice, 10^5^ H22 cells per mouse were inoculated s.c. (7 per group). Five days later, the mice were divided into two groups that received Al(OH)3 and PBS as outlined before.

### Cytokine and chemokine assays

Peritoneal wash fluid and tumor interstitial fluid were collected from mice 3 days after the 1st, 3rd, 6th injections of Al(OH)3 or PBS. For peritoneal wash, mice were i.p. injected with 1 ml of PBS, and the fluid was recollected. For tumor interstitial fluid acquisition, flank tumors were harvested from Balb/c mice, minced and resuspended with 1 ml of RPMI 1640 medium. Cell debris was spun down and the fluids were passed through a 0.2-um syringe filter. All samples were assayed for IL-1β, IL-10, TNFα, IL-6, CCL2, CXCL9 and CCL5 using Mouse Cytokine / Chemokine Magnetic Bead Panel Kit (Milliplex, Darmstadt, Germany) according to the manufacturer’s instructions.

### *In vivo* depletion of neutrophils, CD4^**+**^ and CD8^**+**^ T cells

Briefly, Balb/c mice were injected i.p. with 100 μg functional grade purified anti-Ly6G, anti-mouse CD3, anti-mouse CD4, anti-mouse CD8 or rat IgG2b κ isotype antibody in 200 μl PBS. A maintenance dose of 100 μg antibody was injected i.p. twice a week throughout the entire experimental period to ensure depletion.

### Flowcytometry

5 mice from each tumor-inoculated group were sacrificed 3 days after the 1st, 3rd, 6th injections. Spleens and flank tumors were harvested. Tumor tissue was minced and digested with 4 mg/ml collagenase type IV and 2 mg/ml DNase I (both from Sigma) at 37 °C for 30 min. 5 × 10^5^ cells were added to each well in 96-well, flat-bottom culture plates in 100 μl volume. FITC-conjugated rat anti-mouse CD11b and Pacific Blue-conjugated rat anti-mouse Ly-6G were used to stain neutrophils; FITC-, PE- and APC-labeled anti-mouse CD3e, CD8, CD4 were used to stain CD4^**+**^/CD8^**+**^ T cells. All flowcytometry was performed on LSR Fortessa (BD). Data analysis was done using FlowJo software.

### T cell proliferation

Draining LNs were harvested from tumor-bearing mice. Single cell suspensions were prepared. Cell Proliferation Dye eFluor 670 was dissolved in DMSO as 5 mM stock solutions. For eFluor 670 labeling, lymphocytes were resuspended to 10^7^ cells/ml in PBS and 5 μM eFluor 670 was added to 1 ml aliquots of lymphocytes. Cells were incubated at 20 °C for 10 min and then on ice for 5 min, washed 3 times with culture media. For H22 cell lysate, 3 × 10^8^ H22 cells were collected and resuspended in 2 ml PBS. Three freeze-thaw cycles were performed to obtain cellular lysate. Cells were then centrifuged at 1000 rpm for 3 min. The protein concentration in supernatant was measured by Bradford assay (Bio-Rad), and diluted with PBS to a final concentration of 10 mg/ml. 3 × 10^5^ eFluor 670-labelled lymphocytes were added to each well in 96-well plates in 100 μl volume and stimulated with H22 cell lysate (10 μg/ml) at 37 °C in 5% CO2. Anti-CD3 (1 μg/ml) and anti-CD28 (0.5 μg/ml) were used as positive control. After 72 hour stimulation, lymphocytes were measured by cell surface staining with PE-labeled anti-mouse CD8 mAb and FITC-labeled anti-mouse CD4 mAb, the stained cells were analyzed by FACS. Data were expressed as stimulation index (SI), calculated using the following formula: SI = eFluor 670 tumor cell lysis stimulated / eFluor 670 unstimulated.

### Histology

Tumor-bearing mice were sacrificed and spleen, liver and kidney were excised at times indicated. The tissue was placed in 4% formalin solution. The fixed tissue was bisected longitudinally and embedded in parafilm. 4 to 5 μm sections were made and then stained with H&E. Light microscopy was used to identify the status of necrosis and condensation of nucleus.

### RNA isolation and microarray

Flank tumors were collected 3 days after the 3rd injection. Total RNA and miRNA enrichment procedure were performed using AM1561 mirVana miRNA Isolation Kit without phenol (Ambion). Total RNA was quantified by the NanoDrop ND-2000 (Thermo Scientific) and the RNA integrity was assessed using Agilent Bioanalyzer 2100 (Agilent Technologies). The sample labeling, microarray hybridization and washing were performed based on the manufacturer’s standard protocols. Briefly, total RNA was transcribed to double-stranded cDNA, then synthesized into cRNA and labeled with Cyanine-3-CTP. The labeled cRNA was hybridized onto the microarray (Agilent Mouse Gene Expression 8*60 K, Design ID: 028005). After washing, the arrays were scanned by the Agilent Scanner G2505C. Feature Extraction software (version10.7.1.1, Agilent Technologies) was used to analyze array images to get raw data. Genespring was employed to perform basic analysis with the raw data. In brief, the raw data was normalized with the quantile algorithm. The probes that have at least 100% of the values in any one out of all conditions have flags in “Detected” were chosen for further data analysis. Differentially expressed genes were then identified through fold change as well as P value calculated with t-test. The threshold set for up- and down-regulated genes was a fold change ≥2.0 and a P value ≤0.05. Finally, Hierarchical Clustering was performed to display the distinguishable genes’ expression pattern among samples.

### Mouse neutrophil purification

Whole blood was collected by cardiac puncture using heparinized syringe. The blood was diluted with PBS (1:1) and subjected to a discontinuous Histopaque (Sigma, St. Louis, MO, USA) gradient (1.077 and 1.119). Neutrophils were collected from the 1.077–1.119 interface, lymphocytes and monocytes collected from the plasma-1.077 interface. RBCs were eliminated by hypotonic lysis. The cells were washed twice with PBS and resuspended with RPMI 1640 medium in a final concentration of 10^6^ cells/ml. Neutrophil purity was determined by FACS (>90%).

### *In vitro* killing assay

H22 cells were labelled with Cell Proliferation Dye eFluor 450. The labeled cells (5000/well) were plated on a 96-well in RPMI 1640 medium with 10% FBS. Four hours later, purified neutrophils (10^5^/well) were added to the plated tumor cells and cocultured overnight. Following overnight incubation, the cells were harvested for detection of tumor cell (eFluor 450^**+**^ cells) apoptosis. Apoptosis was recorded using Annexin V-FITC apoptosis detection kit (eBioscience).

### Statistical analysis

Statistical analysis was carried out using GraphPad Prism Software 6.0 and presented as mean ± SEM. An unpaired Student’s t test analysis was used for all data analysis. P < 0.05 indicates statistical significance.

## Additional Information

**How to cite this article**: Wang, B. *et al*. Suppression of established hepatocarcinoma in adjuvant only immunotherapy: alum triggers anti-tumor CD8^+^ T cell response. *Sci. Rep*. **5**, 17695; doi: 10.1038/srep17695 (2015).

## Supplementary Material

Supplementary Information
