# Supplementary material for: Suppression of established hepatocarcinoma in adjuvant only immunotherapy: alum triggers anti-tumor CD8+ T cell response
Source: Sci Rep. 2015 Dec 9;5:17695. doi: 10.1038/srep17695 (PMC4673419; doi:10.1038/srep17695)
Supplement: Supplementary Information [file srep17695-s1.pdf]

Supplemental Information

Suppression of established hepatocarcinoma in adjuvant only immunotherapy: alum triggers anti-tumor $CD8^+$ T cell response

Bo Wang[1], Xuanyi Wang[1,2] , Yumei Wen[1], Jing Fu[3], Hongyang Wang[3], Zhangmei Ma[1], Yan Shi[4*], and Bin Wang[1*]

1. Key Laboratory of Molecular Medical Virology, MOE/MOH, Shanghai Medical College, Fudan University, Shanghai, China

2. Institute of Biomedical Sciences, Fudan University, Shanghai, China

3. International Cooperation Laboratory on Signal Transduction, Eastern Hepatobiliary Surgery Institute/Hospital, and National Center for Liver Cancer, Shanghai, China

4. Institute of Immunology, Department of Basic Medical Sciences, Center for Life Sciences, Tsinghua University, Beijing, China; Department of Microbiology, Immunology and Infectious Diseases, University of Calgary, Calgary, Canada

SFig 1

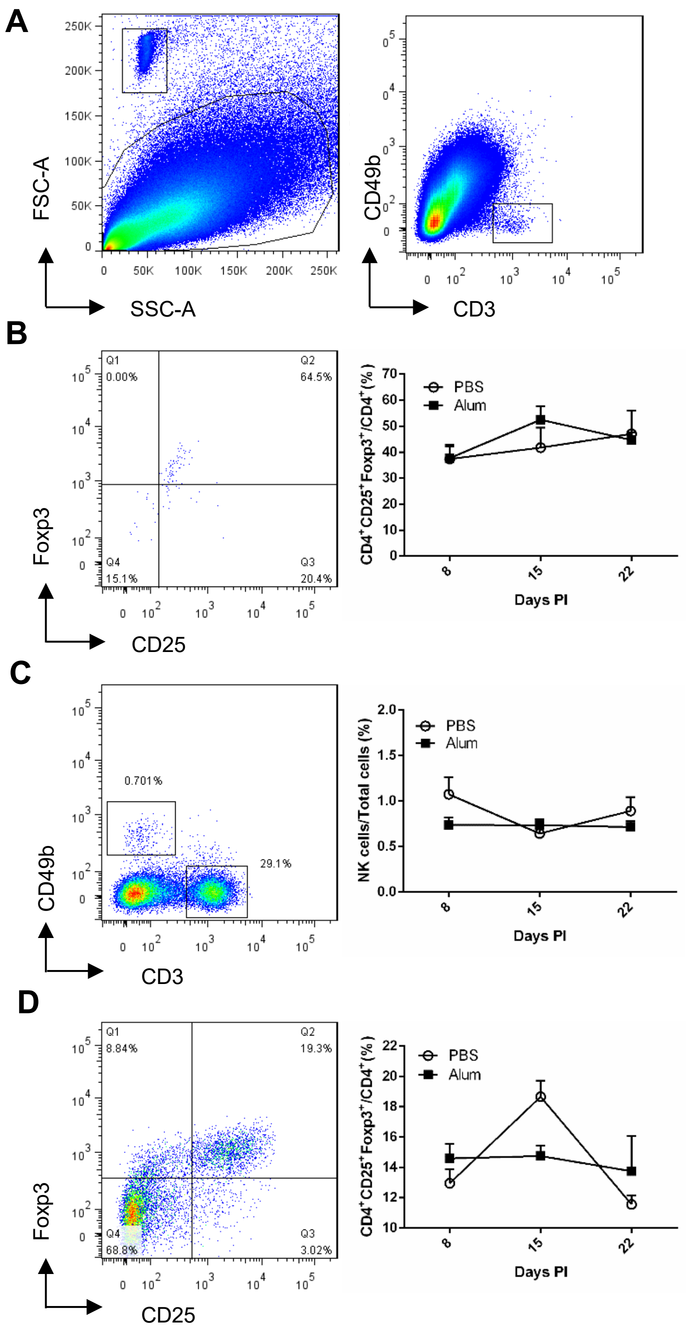

A
250K
200K
150K
100K
50K
0
FSC-A
0
50K
100K
150K
200K
250K
SSC-A
CD49b
CD3
B
Q1
0.00%
Q2
64.5%
Q4
15.1%
Q3
20.4%
Foxp3
CD25
PBS
Alum
CD4+CD25+Foxp3+/CD4+(%)
Days PI
C
0.701%
29.1%
CD49b
CD3
NK cells/Total cells (%)
Days PI
D
Q1
8.84%
Q2
19.3%
Q4
68.8%
Q3
3.02%
Foxp3
CD25
CD4+CD25+Foxp3+/CD4+(%)
Days PI

SFig 2

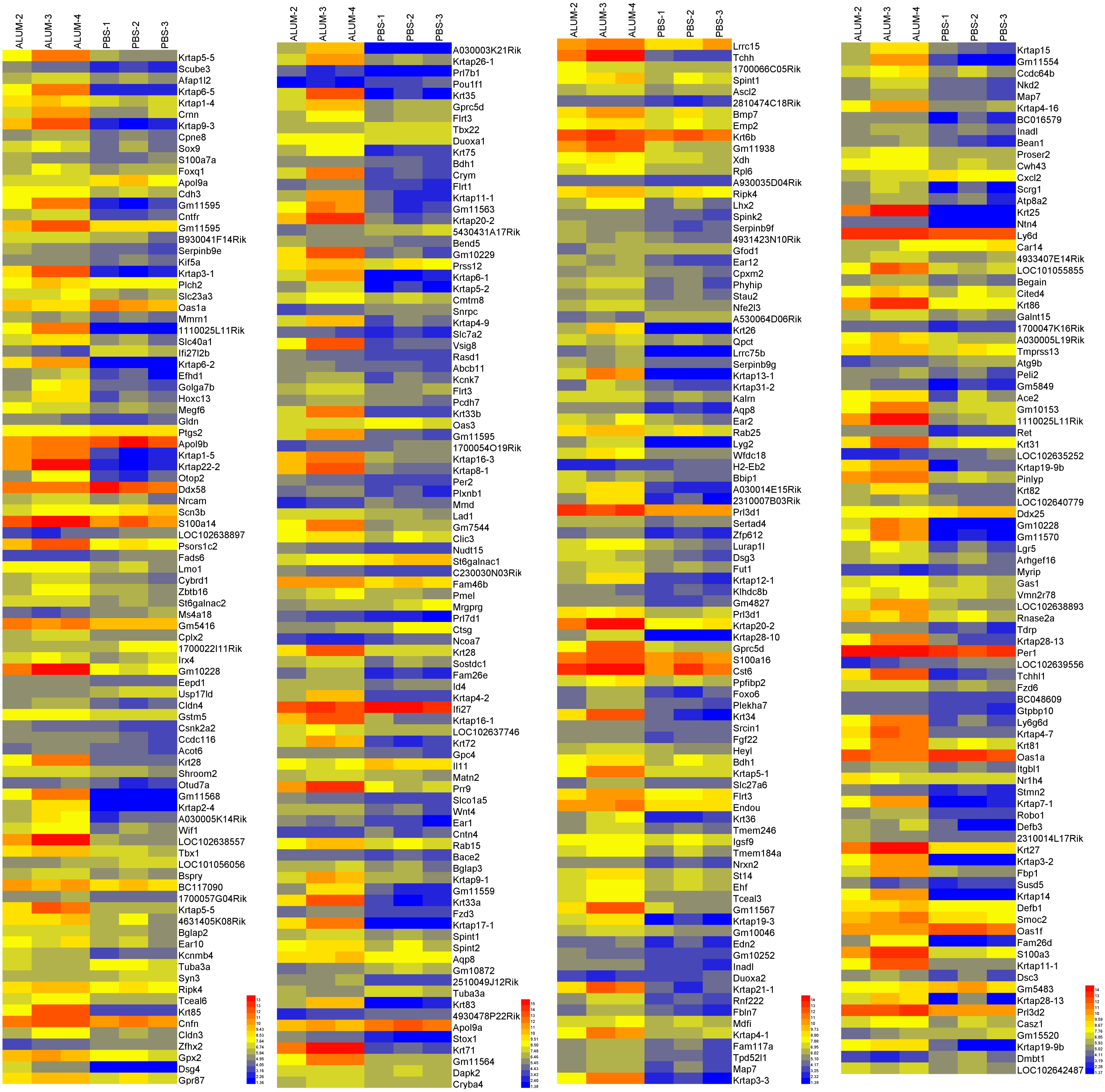
ALUM-2
ALUM-3
ALUM-4
PBS-1
PBS-2
PBS-3
Krtap5-5
Scube3
Afap1l2
Krtap6-5
Krtap1-4
Crnn
Krtap9-3
Cpne8
Sox9
S100a7a
Foxq1
Apol9a
Cdh3
Gm11595
Cntfr
Gm11595
B930041F14Rik
Serpinb9e
Kif5a
Krtap3-1
Plch2
Slc23a3
Oas1a
Mmrn1
1110025L11Rik
Slc40a1
Ifi27l2b
Krtap6-2
Efhd1
Golga7b
Hoxc13
Megf6
Gldn
Ptgs2
Apol9b
Krtap1-5
Krtap22-2
Otop2
Ddx58
Nrcam
Scn3b
S100a14
LOC102638897
Psors1c2
Fads6
Lmo1
Cybrd1
Zbtb16
St6galnac2
Ms4a18
Gm5416
Cplx2
1700022I11Rik
Irx4
Gm10228
Eepd1
Usp17ld
Cldn4
Gstm5
Csnk2a2
Ccdc116
Acot6
Krt28
Shroom2
Otud7a
Gm11568
Krtap2-4
A030005K14Rik
Wif1
LOC102638557
Tbx1
LOC101056056
Bspry
BC117090
1700057G04Rik
Krtap5-5
4631405K08Rik
Bglap2
Ear10
Kcnmb4
Tuba3a
Syn3
Ripk4
Tceal6
Krt85
Cnfn
Cldn3
Zfhx2
Gpx2
Dsg4
Gpr87
ALUM-2
ALUM-3
ALUM-4
PBS-1
PBS-2
PBS-3
A030003K21Rik
Krtap26-1
Prl7b1
Pou1f1
Krt35
Gprc5d
Flrt3
Tbx22
Duoxa1
Krt75
Bdh1
Crym
Flrt1
Krtap11-1
Gm11563
Krtap20-2
5430431A17Rik
Bend5
Gm10229
Prss12
Krtap6-1
Krtap5-2
Cmtm8
Snrpc
Krtap4-9
Slc7a2
Vsig8
Rasd1
Abcb11
Kcnk7
Flrt3
Pcdh7
Krt33b
Oas3
Gm11595
1700054O19Rik
Krtap8-1
Per2
Plxnb1
Mmd
Lad1
Gm7544
Clic3
Nudt15
St6galnac1
C230030N03Rik
Fam46b
Pmel
Mrgprg
Prl7d1
Ctsg
Ncoa7
Krt28
Sostdc1
Fam26e
Id4
Krtap4-2
Ifi27
Krtap16-1
LOC102637746
Krt72
Gpc4
Il11
Matn2
Prr9
Slco1a5
Wnt4
Ear1
Cntn4
Rab15
Bace2
Bglap3
Krtap9-1
Gm11559
Krt33a
Fzd3
Krtap17-1
Spint1
Spint2
Aqp8
Gm10872
2510049J12Rik
Tuba3a
Krt83
4930478P22Rik
Apol9a
Stox1
Krt71
Gm11564
Dapk2
Cryba4
ALUM-2
ALUM-3
ALUM-4
PBS-1
PBS-2
PBS-3
Lrrc15
Tchh
1700066C05Rik
Spint1
Ascl2
2810474C18Rik
Bmp7
Emp2
Krt6b
Gm11938
Xdh
Rpl6
A930035D04Rik
Ripk4
Lhx2
Spink2
Serpinb9f
4931423N10Rik
Gfod1
Ear12
Cpxm2
Phyhip
Stau2
Nfe2l3
A530064D06Rik
Krt26
Qpct
Lrrc75b
Serpinb9g
Krtap13-1
Krtap31-2
Kalrn
Aqp8
Ear2
Rab25
Lyg2
Wfdc18
H2-Eb2
Bbip1
A030014E15Rik
2310007B03Rik
Prl3d1
Sertad4
Zfp612
Lurap1l
Dsg3
Fut1
Krtap12-1
Klhdc8b
Gm4827
Prl3d1
Krtap20-2
Krtap28-10
Gprc5d
S100a16
Cst6
Ppfibp2
Foxo6
Plekha7
Krt34
Srcin1
Fgf22
Heyl
Bdh1
Krtap5-1
Slc27a6
Flrt3
Endou
Krt36
Tmem246
Igsf9
Tmem184a
Nrxn2
St14
Ehf
Tceal3
Gm11567
Krtap19-3
Gm10046
Edn2
Gm10252
Inadl
Duoxa2
Krtap21-1
Rnf222
Fbln7
Mdfi
Krtap4-1
Fam117a
Tpd52l1
Map7
Krtap3-3
ALUM-2
ALUM-3
ALUM-4
PBS-1
PBS-2
PBS-3
Krtap15
Gm11554
Ccdc64b
Nkd2
Map7
Krtap4-16
BC016579
Inadl
Bean1
Proser2
Cwh43
Cxcl2
Scrg1
Atp8a2
Krt25
Ntn4
Ly6d
Car14
4933407E14Rik
LOC101055855
Begain
Cited4
Krt86
Galnt15
1700047K16Rik
A030005L19Rik
Tmprss13
Atg9b
Peli2
Gm5849
Ace2
Gm10153
1110025L11Rik
Ret
Krt31
LOC102635252
Krtap19-9b
Pinlyp
Krt82
LOC102640779
Ddx25
Gm10228
Gm11570
Lgr5
Arhgef16
Myrip
Gas1
Vmn2r78
LOC102638893
Rnase2a
Tdrp
Krtap28-13
Per1
LOC102639556
Tchhl1
Fzd6
BC048609
Gtpbp10
Ly6g6d
Krtap4-7
Krt81
Oas1a
Itgbl1
Nr1h4
Stmn2
Krtap7-1
Robo1
Defb3
2310014L17Rik
Krt27
Krtap3-2
Fbp1
Susd5
Krtap14
Defb1
Smoc2
Oas1f
Fam26d
S100a3
Krtap11-1
Dsc3
Gm5483
Krtap28-13
Prl3d2
Casz1
Gm15520
Krtap19-9b
Dmbt1
LOC102642487

Supplemental Figure Legend

SFig 1. Balb/c mice were inoculated s.c. with H22 cells and treated with $Al(OH)_3$ or PBS. Tumor and draining LNs were harvested from tumor-bearing mice on day 3 after the 1st, 3rd and 6th injection. Single cell suspensions were prepared. T, NK and Treg cells were identified using CD3, CD49b (DX5), FoxP3/CD25 antibodies respectively and percentages of these cells are shown. The percentages of NK cells in total cells of tumor (A) or draining LNs (C) were analyzed by FACS, as were the percentages of Treg cells in $CD4^+$ T cells of tumor (B) or draining LNs (D). Notice that there was essentially no detectable NK population in A.

SFig 2. As in Fig 4A, heatmap for all transcripts analyzed is shown
